# Modeling the Development of Audiovisual Cue Integration in Speech Perception

**DOI:** 10.3390/brainsci7030032

**Published:** 2017-03-21

**Authors:** Laura M. Getz, Elke R. Nordeen, Sarah C. Vrabic, Joseph C. Toscano

**Affiliations:** Department of Psychology, Villanova University, Villanova, PA 19085, USA; enordeen@villanova.edu (E.R.N.); svrabic@villanova.edu (S.C.V.); joseph.toscano@villanova.edu (J.C.T.)

**Keywords:** speech perception, speech development, multimodal representations, audiovisual cues, statistical learning, mixture of Gaussians, cue weighting

## Abstract

Adult speech perception is generally enhanced when information is provided from multiple modalities. In contrast, infants do not appear to benefit from combining auditory and visual speech information early in development. This is true despite the fact that both modalities are important to speech comprehension even at early stages of language acquisition. How then do listeners learn how to process auditory and visual information as part of a unified signal? In the auditory domain, statistical learning processes provide an excellent mechanism for acquiring phonological categories. Is this also true for the more complex problem of acquiring audiovisual correspondences, which require the learner to integrate information from multiple modalities? In this paper, we present simulations using Gaussian mixture models (GMMs) that learn cue weights and combine cues on the basis of their distributional statistics. First, we simulate the developmental process of acquiring phonological categories from auditory and visual cues, asking whether simple statistical learning approaches are sufficient for learning multi-modal representations. Second, we use this time course information to explain audiovisual speech perception in adult perceivers, including cases where auditory and visual input are mismatched. Overall, we find that domain-general statistical learning techniques allow us to model the developmental trajectory of audiovisual cue integration in speech, and in turn, allow us to better understand the mechanisms that give rise to unified percepts based on multiple cues.

## 1. Introduction

There is no question that speech perception is a multimodal process (see [1,2] for reviews). In face-to-face conversations, the listener receives both visual information from the speaker’s face (e.g., their lips, teeth, tongue, and non-mouth facial features) and acoustic signals from the speaker’s voice. In order to use these two sources of information, listeners must combine auditory and visual cues into an integrated percept during spoken language comprehension. A number of studies show that the reliable co-occurrence of synchronous and highly redundant visual and auditory cues supports this ability, leading to accurate speech comprehension by adults [3,4], especially in cases where the auditory signal is degraded due to background noise [5,6,7,8,9]. Mismatching auditory and visual information also influences speech perception, as shown in the McGurk effect [10,11]: listening to the spoken syllable /ba/ while simultaneously watching the visual movements for /ga/ often results in the illusory perception of /da/. The McGurk effect provides clear evidence that visual information is involved in speech perception even when the auditory signal is perfectly intelligible [12].

How does the ability to perceive a unified audiovisual percept emerge over the course of development? Are the same developmental processes involved in infants’ acquisition of phonological categories via acoustic cues used for acquisition of categories based on visual cues? In this paper, we aim to address these questions by presenting a model of phonetic category acquisition that is trained on data derived from phonetic analyses of visual and auditory speech cues for stop consonants. The model uses the same statistical learning processes available to human infants; thus, in principle, it allows us to determine whether such processes are sufficient for the acquisition of integrated audiovisual percepts.

### 1.1. Audiovisual Speech Development in Infants

Despite the fact that multimodal inputs benefit speech perception in adults, it is not clear that integrated audiovisual inputs equally benefit infants’ speech perception [13] or whether infants even form integrated audiovisual perceptual representations. This does not mean that infants do not use visual speech cues; indeed, research shows that infants can detect characteristics of both visual and auditory speech even before they can speak [14]. Infants can also detect audiovisual temporal asynchrony in isolated syllables and continuous speech streams [15,16] and prefer to listen to fluent, synchronized speech [17]. Thus, infants’ are able to track the co-occurrence of temporally-aligned cues, an ability that may provide a mechanism for later development of integrated perceptual representations.

There is some evidence that infants can detect cases where phonemic information is congruent between faces and voices. In several studies, infants were habituated with two side-by-side faces silently mouthing different phonemes that are reliably distinguished by visual cues, such as /b/ and /d/. The infants were then presented with a simultaneous auditory stimulus that matched one of the faces and their looking time to the two faces was measured. Four- to five-month-old [18,19,20], two-month old [21], and even newborn [22] infants consistently look longer at the matching face than the mismatched face, providing evidence that infants can rapidly learn to track these phonological co-occurrences.

Research is more mixed as to when infants can detect cases where fluent (i.e., continuous) speech information matches between faces and voices. Using a similar paradigm to that described above, one study found that four- to eight-month-old infants did not show a preference for a face that was temporally synchronized and matched with the articulation of an auditory stimulus, whereas 12- to 14-month-olds did show such a preference [23]. However, a different study found that eight-month-old infants did show a preference for congruent speech patterns across modalities, even with a visual signal that consisted only of animated markers on a talking face and with a low-pass filtered auditory signal [24]. A similar study was done with newborns, who also showed a preference for the matching face and voice [25]. Interestingly, infants can also match a multi-syllable auditory signal with the correct articulating face when the sound is sine wave speech (SWS, see [26]), suggesting that infants may be relying on cues that are not necessarily phonetic to match auditory and visual speech [27].

Studies examining the McGurk effect in infants provide even more ambiguous results. Several studies with infants as young as four months found that their pattern of illusory phoneme detection was similar to that of adults [13,28]. However, other studies have found different results in male and female infants depending on the experimental design [29,30], showing that the effect is not as strong or consistent as in adults. Therefore, the literature suggests that infants are sensitive to cases of temporal and phonological audiovisual synchrony, suggesting a possible innate audiovisual integration skill. However, it is not clear that this sensitivity assists them with the development and mastery of speech perception [31], as it does for adults.

### 1.2. Audiovisual Speech Development in Older Children

Research with preschoolers and school-age children provides further evidence that children do not yet fully integrate multi-modal speech cues, and provides insights into the developmental time-course of this process. Children weight visual cues less, relative to adults, in speech categorization tasks involving cues from both modalities [32]. In addition, a majority of research using McGurk-like paradigms (i.e., comparisons between congruent and incongruent audiovisual stimuli) show that children rely less on visual cues than adults. A number of studies find that the influence of the illusory phoneme varies as a direct function of age, with children aged four-to nine-years-old largely choosing a response consistent with the auditory stimulus and children ages 10–18 more often choosing the illusory phoneme (i.e., the response consistent with the visual stimulus; [33,34,35,36,37]). This developmental time-course has also been observed in a recent event-related potential (ERP) study [38]. In adult subjects, the amplitude of the N1 and P2 ERP components is reduced in the presence of incongruent audiovisual stimuli, relative to auditory-only stimuli, suggesting that visual speech information affects auditory perceptual representations. Knowland et al. [38] found that this amplitude reduction for audiovisual stimuli emerges over development, occurring around the same age as corresponding behavioral effects for audiovisual speech. Research also shows that younger children do not benefit as much as adults from the addition of visual cues in noisy environments. Studies using noise-vocoded sentences [39] and low auditory signal-to-noise (SNR) ratios [31,40,41] have found that children do not fully utilize visual speech cues to enhance speech perception when the auditory signal is degraded.

Several possible explanations for the lack of integration have been offered. One explanation is that young children (i.e., four- to six-year-olds) have poor lip-reading ability. Indeed, one study found a positive correlation between lip-reading ability and the size of the visual contribution to audiovisual speech perception [32]. Another explanation may be that young children (i.e., three- to five-year-olds) lack the necessary experience correctly producing the phoneme sounds in order to integrate this information during comprehension. In support of this explanation, one study found that preschool children who make phoneme articulation errors for consonant confusions are less influenced by visual cues than those children who correctly produce the consonants [42]. A third possibility is that audiovisual speech allows for predictive coding (because the visual information normally arrives first), which can speed up processing in adult subjects [43]; however, it is unclear how the ability to use this information might change over development.

However, not all results suggest the same developmental pattern. One study found a significant influence of visual speech in four year-olds and 10- to 14-year-olds, but not five- to nine-year-olds, proposing a temporary loss in sensitivity due to a reorganization of phonological knowledge during early school years [44]. In contrast, other studies have shown an influence of visual speech in children as young as six-year-olds on detection, discrimination, and recognition audiovisual speech tasks [45].

In general, the evidence reported here seems to suggest that although infants show early sensitivity to both auditory and visual speech information, the accuracy of using and combining information from multiple modalities to acquire phonological categories improves throughout childhood [12,38]. In addition, data on the developmental time-course of this process suggest that, in general, children rely more heavily on auditory speech cues than visual ones, relative to adults [32]. Our goal here is to provide a mechanism that can explain how unified percepts are formed over development, and why these developmental patterns emerge.

### 1.3. Acquisition of Auditory Phonological Categories

There is an extensive literature on children’s acquisition of phonological distinctions in the auditory domain that may help shed light on the processes involved in the acquisition of multimodal phonological categories. Classic studies on infant speech sound discrimination [46,47] suggest differences between infant and adult speech sound discrimination. In some cases this manifests as increased sensitivity to non-native speech sound contrasts (e.g., for stop consonants; [47]), while in other cases infants show decreased sensitivity to acoustic differences (e.g., for fricative distinctions; [48]). Thus, there is evidence for a perceptual reorganization process over development; over the first year or two, infants learn phonological representations that are specific to their native language.

How do infants do this? Consider that any learning mechanism that seeks to explain the process of phonological acquisition faces a significant challenge: infants appear to learn their native-language speech sound categories without explicit feedback about how acoustic cues map onto different phonemes. They must sort out which acoustic cues are used in their native language and how many phoneme categories their language has along any given phonological feature dimension (e.g., English has two voicing categories, while Thai has three, [49]). Infants also process input stochastically, updating their representations on a trial-by-trial basis.

These factors point towards a need for an *unsupervised* learning process that allows for the gradual acquisition of speech sound categories over time. Statistical learning provides one possible mechanism for this. For example, previous work has demonstrated that infants can track the distributional statistics of phoneme transitions (i.e., the probability that a phoneme occurs within or at the end of a word) and can use this information to segment words [50] in a discrimination task.

The same process can be applied to the acquisition of phonological categories based on the distributional statistics of acoustic-phonetic cues. For example, English has a two-way voicing distinction between unaspirated (typically referred to as “voiced”) and aspirated (typically referred to as “voiceless”) stop consonants. Word-initially, this distinction is primarily signaled by differences in voice onset time (VOT), a reliable cue that is used to mark voicing distinctions in many languages. The distribution of VOT values in English tends to form two clusters, one corresponding to voiced and one to voiceless consonants. Thus, the distributional statistics for VOT contain information needed to map specific cue-values onto phonemes.

Infants appear to be sensitive to this statistical information [51]. Infants exposed to speech sounds that form a bimodal distribution (reflecting a two-category phonological distinction) will discriminate pairs of sounds that correspond to the two categories, whereas infants exposed to a unimodal distribution (reflecting an acoustic cue that is not used to provide a meaningful contrast in the language) show poorer discrimination. This is true even though the overall likelihood of hearing the specific tokens is the same in both conditions. Thus, infants are able to track the distributional statistics of specific acoustic cues, which can then be used to acquire phonological categories.

McMurray et al. [52] implemented this unsupervised statistical learning mechanism in a Gaussian mixture model (GMM) of speech development. This approach provides a learning mechanism that requires few assumptions about the knowledge available to infants, as it does not require the modeler to specify the number of phonological categories or their statistical properties *a priori*, and it involves learning categories using the same iterative statistical learning procedure available to infants. In their simulations, McMurray et al. demonstrated that phoneme categories based on VOT distinctions can be learned via statistical learning and winner-take-all competition. On each training trial, the phoneme category with the highest likelihood for the current input is updated, thus eliminating unnecessary Gaussians and eventually converging on the appropriate two-category voicing distributions. The model successfully accounts for different developmental trajectories, showing both overgeneration-and-pruning effects and phonological enhancement effects observed in human infants [47,48].

### 1.4. Cue Weighting in Speech

This statistical learning approach has also been extended to the acquisition of multiple acoustic cues. The speech signal contains redundant cues that provide information about specific phonological contrasts [53,54,55]. For example, Lisker [55] lists 16 cues to word-medial voicing in English. Thus, we must also consider how multiple acoustic cues are combined over the course of development. This can be extended to the questions about combining auditory and visual speech cues as well.

A few studies have examined how children combine multiple acoustic cues and how their cue weights change over time. Data on fricative classification (/f/ vs. /θ/ and /s/ vs. /ʃ/ distinctions) suggests that infants initially weight dynamic acoustic cues (formant transitions) more than static spectral differences (mean frication frequency) [56]. However, the relative weights of these cues may be specific to those phonemes, as different patterns emerge for the relative weight of temporal and spectral cues to stop consonant voicing (/d/ vs. /t/; [57,58]). In both cases, however, it is clear that children’s cue weights are not the same as adults’ weights, suggesting some process that involves a change in cue weights over the course of development. This observation is similar to the work described above showing that children tend to weight visual speech cues less than adults overall.

The computational modeling approach described above has also been applied to the problem of the acquisition of multiple acoustic cues. Toscano and McMurray [59] extended the GMM introduced by McMurray et al. [52] to allow the model to learn an arbitrary number of cues for any given phonological feature, combining them into a unified perceptual representation. In their simulations, they demonstrate that VOT and vowel length (VL, a secondary cue to voicing) are weighted approximately by their reliability. Weighting-by-reliability (an approach used in our model as well) derives from well-established effects in visual perception describing how observers combine cues to depth: cues are weighted inversely to the variance in the estimates they provide (i.e., cues that provide more consistent depth estimates are weighted higher). Thus, the weight of a cue can be described as the inverse of its variance,

(1) $$w=\frac{1}{\sigma^{2}}$$

where *σ* is the standard deviation of the estimates provided by that cue. This metric can be applied to both multiple visual cues [60] and cross-modal integration (i.e., combining visual and haptic cues; [61]), and it describes an optimal method of weighting cues given their statistical reliability.

Although this approach works well for unimodal dimensions, it would not accurately describe cue reliability for a multimodal dimension where variability arises from naturally-occurring differences between category exemplars (as opposed to variability due to noise in the cue estimates, as in the models described above). The is the case for speech sounds, which are structured by phonological categories (e.g., voiced and voiceless VOT categories). If we ignore this category structure and apply Equation (1), we will obtain a *lower* cue weight for categories that are further spread apart, all other things being equal. This is precisely the opposite of how the metric should weight this cue. Toscano and McMurray [59] present a different metric, implemented in a weighted Gaussian mixture model (WGMM), that applies the weighting-by-reliability principle to perceptual dimensions that contain a category structure, as acoustic and visual cues in speech do. For a two-way distinction (e.g., /b/ vs. /d/), the cue weighting metric is

(2) $$w=\frac{\phi_{m}\phi_{n}{\left(\mu_{m}-\mu_{n}\right)}^{2}}{\sigma_{m}\sigma_{n}}$$

where *μ* is the mean of each category, *σ* is its standard deviation, and *ϕ* is the category’s frequency of occurrence in the language (allowing the model to account for phoneme categories that are not equally likely). *m* and *n* correspond to values for the two phoneme categories. Thus, if the means of two categories are far apart, the cue dimension will be weighted higher; if the variances within each category are high, the cue will be weighted lower. Toscano and McMurray [59] demonstrate that this cue weighing metric captures the way that listeners combine multiple acoustic cues in speech.

Critically, they also demonstrate that the developmental process matters. When cue weights are set strictly by their statistical reliability, the model underestimates the weight of the secondary cue (VL). However, when the model is allowed to *learn* the cue weights via an unsupervised statistical learning process, it arrives at category representations that more closely reflect those of adult listeners.

Other models describing acoustic cue-integration rely on similar principles [62,63,64], and several models have been proposed to explain either perceptual integration more generally [65] or audiovisual cue-integration in speech specifically [66,67,68,69]. Although previous audiovisual cue-integration models have not focused on describing the underlying developmental mechanisms, they still offer a number of insights.

Perhaps the most influential model in the audiovisual speech domain has been Massaro and colleagues’ fuzzy logical model of perception (FLMP) [69], which extended the original implementation that was designed to handle multiple acoustic cues in speech [62]. FLMP maps continuously-valued cues probabilistically onto phonological categories via a three-step process: (1) feature evaluation, the process of encoding features as initial perceptual representations; (2) prototype matching, which involves mapping feature-values onto stored category prototypes; and (3) pattern classification, which involves determining which prototype (category) best matches the input.

FLMP can also be fit to perceptual data from individual subjects performing audiovisual speech categorization tasks. While it provides a good fit to these data when cues are treated as independent [69], as they are in the WGMM used here, there is debate about whether similar results can be achieved with fewer model parameters (e.g., in the early maximum likelihood estimation model; [66]) or when cues are combined at a pre-categorization stage (as in the pre-labeling integration model; [68]). Recent work has also aimed to incorporate the weighting-by-reliability perspective to explain audiovisual speech perception, taking the category structure of the acoustic and visual cue dimensions into account [67], using a cue weighting metric similar to the one used in the WGMM (using the variance across the cue dimension, rather than the within-category variances, in the denominator). Overall, modeling work in audiovisual speech perception has generally supported the view that listeners combine cues probabilistically based on the amount of information provided by each cue. However, this work has not focused on describing the developmental mechanisms that give rise to these integrated percepts, which we plan to do here.

### 1.5. Approach

The main goal of the present study was to address two limitations of previous models: (a) previous audiovisual integration models have not sought to describe the developmental mechanisms that give rise to the changes in cue-weighting observed between children and adults (though they have sought to fit models to data from both children and adults; [32]); and (b) previous cue integration models that do describe development (e.g., WGMM) [59] have focused only on acoustic cues; they have not demonstrated that unsupervised statistical learning is sufficient to acquire these types of audiovisual representations.

Is it the case that listeners also use statistical learning for the complex problem of acquiring audiovisual speech categories, which require the learner to integrate information from multiple modalities? Can children learn to appropriately weight cues from different senses according to their reliability? Here, we ask whether WGMMs can be used to simulate the acquisition of phonological distinctions for voiced stop consonants (/b/, /d/, and /ɡ/) and capture the changes in cue weights for multiple auditory and visual cues observed over development.

The data used in our simulations consists of distributions of acoustic and visual cue measurements from the Audio-Video Australian English Speech (AVOZES) data corpus [70,71] as reported in Goecke [72]. The AVOZES corpus comprises 20 native speakers of Australian English (10 female and 10 male speakers) recorded producing phonemes using a non-intrusive 3D lip tracking algorithm. From the recordings, they extracted a number of auditory speech parameters (fundamental frequency F0, formant frequencies F1, F2, F3, and RMS energy) and visual speech parameters (mouth width, mouth height, protrusion of the upper lip, protrusion of the lower lip, and relative teeth count).

Our simulations examined four of these cues (two visual, and two acoustic) that we predicted would be the most reliable and uncorrelated with each other: (1) mouth width (*MW*; defined as the 3D distance from lip corner to lip corner); (2) mouth height (*MH*; 3D distance from the midpoint of upper lip to the midpoint of lower lip); (3) F2 onset (*F2*), and F3 onset (*F3*). F2 and F3 are canonical acoustic cues to place of articulation [73], and MH and MW were predicted to be the most orthogonal to each other, given the visual cues in the AVOZES corpus (since they are measures of orthogonal dimensions in space). Although there are many more phonetic cues in speech for any given phonological dimension [55], the subset of cues we use here are informative and approximate the categorization functions produced by human subjects, as evidenced by the identification curves produced by the model. In theory, we could add any number of cues to the simulations; the model should learn which cues are uninformative because their cue-weights should be near zero.

The purpose of our current approach was to simulate the developmental process of acquiring phonological categories from auditory and visual cues, asking whether simple statistical learning approaches are sufficient for learning multi-modal representations. Further, we use this information to explain audiovisual speech perception in adult perceivers, including cases where auditory and visual inputs are redundant and cases where the inputs are mismatched. Note that, unlike many of the previous studies aimed at modeling audiovisual cue-integration described above, our approach here is not to fit model parameters to specific subjects’ data, but rather to determine whether a model trained on speech input corresponding to what a human infant might hear will show the same developmental time-course and cue-weighting strategies as humans. This modeling approach is in the same spirit as connectionist simulations, where the goal is to uncover the underlying mechanisms that give rise to behavioral phenomena.

**Figure 1:**
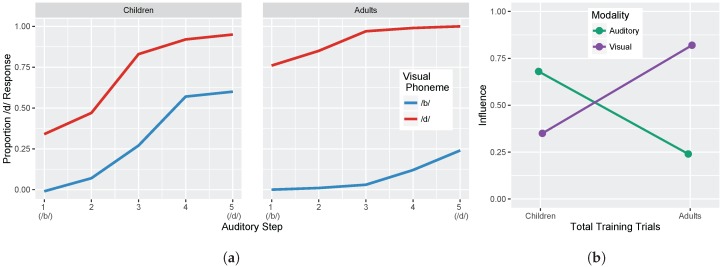
(**a**) Schematic illustration of predicted results showing the observed proportion of /d/ responses as a function of five auditory and two visual steps (based on [32]). Large differences are expected over the course of development. Children respond largely based on auditory information (i.e., their proportion of /d/ responses is similar regardless of visual information). Conversely, adults respond largely based on visual information (i.e., their proportion of /d/ responses is similar regardless of auditory information). The amount of *auditory* influence is shown by the difference in response proportion between one end of the auditory continuum to the other (e.g., from one end of the blue line to the other). The amount of *visual* influence is shown by the difference in response proportion between the two lines (e.g., from the blue line to the red line at the same auditory step); (**b**) Schematic illustration showing the change in auditory and visual influence over development: visual influence increases whereas auditory influence decreases (see Equations (3) and (4)).

We conducted two sets of simulations with the WGMM trained on the four cues, testing the model at five different points during training. At each testing point during development, we measured the relative use of auditory and visual cues to see whether the model shows an increase in reliance on visual cues over the course of development (see Figure 1a). This is similar to the approach used previously by Massaro and colleagues [32,35,62] to examine changes in the proportion of /b/ and /d/ responses to audiovisual stimuli in a forced-choice experiment over the course of development. For children, the proportion of /d/ responses is largely based on *auditory* information. This is shown in schematic Figure 1a (“Children” panel) by the closeness of red and blue lines. Additionally, each of the identification functions span from largely /b/ to largely /d/ responses across the auditory continuum. Later in development, the pattern changes, and the proportion of /d/ responses is largely based on the *visual* information. This is shown in Figure 1a (“Adults” panel) by the spread of the red and blue lines. Additionally, individual identification functions no longer span the entire range of responses across the auditory continuum. We predict a similar pattern of developmental change for the model’s responses.

We also focused more closely on the differences between responses to congruent and incongruent audiovisual stimuli by examining the two endpoint steps from the auditory and visual continua. These proportions were derived from the full identification functions (Figure 1a). The amount of *auditory* influence when the modalities provide incongruent information is shown by the difference in response proportion between the endpoints of the auditory continuum for each visual phoneme, values of which are then averaged together:

(3) $$A_{influence}=\frac{\left(A_{d}V_{b}-A_{b}V_{b}\right)+\left(A_{d}V_{d}-A_{b}V_{d}\right)}{2}$$

where $A_{d}V_{b}$ is the auditory /d/ paired with the visual /b/; the same notation is used for the other stimulus combinations. The amount of *visual* influence when the two modalities provide incongruent information is shown by the difference in response proportion between the visual phonemes at the two auditory continuum endpoints, values of which are then averaged together:

(4) $$V_{influence}=\frac{\left(A_{b}V_{d}-A_{b}V_{b}\right)+\left(A_{d}V_{d}-A_{d}V_{b}\right)}{2}$$

Given previous results [32,33,34,35,36,37], we predicted that the model should show that visual influence is small early in development and larger later in development, whereas auditory influence is large early in development and smaller later in development (see Figure 1b).

In Simulation 1, we examine the acquisition of /b/ vs. /d/ distinctions, and in Simulation 2, we examine /b/ vs. /ɡ/ distinctions. In both cases, the difference between the bilabial consonant and the other phoneme should be conveyed reliably by visual information in addition to being cued by auditory differences. Note that we did not train the model on all three phonemes simultaneously because the acoustic and visual cues used here do not reliably distinguish /d/ and /ɡ/ (see Discussion for further consideration of this point).

## 2. Method

The model is based on Toscano and McMurray’s [59] Gaussian mixture model (GMM) of cue integration, implemented in MATLAB. A brief overview of the model architecture and learning algorithms are given here, including modifications from the original implementation and the details of the procedures used to train the model for the current simulations. Critically, this approach does not require the modeler to specify (1) the number of phonological categories; (2) their statistical properties; or (3) the weight that should be assigned to each cue. This information must be acquired by the model using the same types of unsupervised statistical learning mechanisms available to human infants [51].

### 2.1. Model Architecture

**Figure 2:**
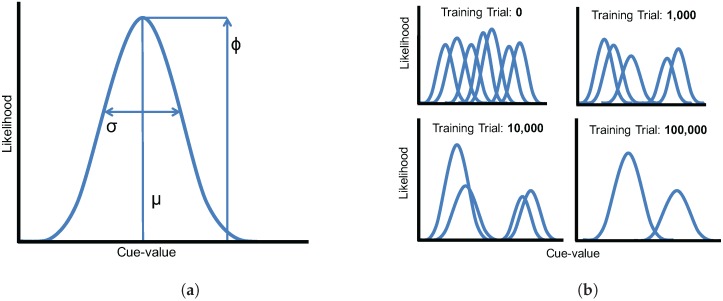
(**a**) Parameters of a Gaussian distribution used in the mixture model. Each distribution is defined by its likelihood (*ϕ*), its mean (*μ*), and its standard deviation (*σ*); (**b**) A schematic illustration of the model training procedure. Mixture components that are not needed to capture the category structure of the language are eliminated over the course of training (i.e., their likelihood parameters, *ϕ*, are reduced to near zero).

The model represents phonological categories as parametric Gaussian distributions. Each acoustic and visual cue dimension is represented as a mixture of Gaussians, with *K* components (i.e., potential categories) in each mixture. To map a cue (*i*) onto a category (*j*), we compute the likelihood of a specific cue-value (*x*) for each Gaussian in the mixture (see Figure 2a),

(5) $$G_{j}\left(x_{i}|\mu_{j},\sigma_{j},\phi_{j}\right)=\frac{\phi_{j}}{\sqrt{2\pi \sigma_{j}^{2}}}exp\left(\frac{{\left(x_{i}-\mu_{j}\right)}^{2}}{2\sigma_{j}^{2}}\right),$$

where *x* is the cue-value, *μ* is the mean for that Gaussian, *σ* is its standard deviation, and *ϕ* is its frequency of occurrence. The overall likelihood for a particular cue is given by the sum of the Gaussians in the mixture,

(6) $$M\left(x\right)=\sum_{j=1}^{K}G_{j}\left(x\right)$$

where *K* represents the number of Gaussians in the mixture. In all of the simulations presented here, K=50, meaning that each mixture could represent up to 50 possible phonological categories. This value is much greater than the true number of categories along each dimension, but this is intentional: The model must learn how many categories there are along each dimension rather than having this information given *a priori*. This is the same developmental challenge faced by human infants learning phoneme categories based on acoustic and visual inputs, since languages vary in the number of categories along each phonological dimension, in addition to their statistical properties. As the model learns, it “prunes away” unnecessary components to arrive at a category representation that reflects the structure of the phonological categories of the language.

In addition to Gaussian mixtures for each acoustic and visual cue, the model also includes a combined mixture (also with K=50 components) that integrates inputs from each cue. This combined representation corresponds to the more abstract phonological dimension that the model is being trained on (e.g., stop consonant place of articulation). In our simulations, this representation corresponds to the model’s integrated audiovisual percept.

### 2.2. Cue Weighting

During training, inputs from each cue are presented to the model, the parameters of the cue-level mixture are updated, and inputs are passed up to the integrated representation. Because the units along each cue dimension differ (i.e., F2 and F3 are measured in Hertz; MW and MH are measured in millimeters), cue-values are normalized prior to being presented to the integrated mixture, using a measure of central tendency,

(7) $$c=\frac{\sum_{j=1}^{K}\mu_{j}\phi_{j}}{\sum_{j=1}^{K}\phi_{j}},$$

and variability,

(8) $$v=\frac{\sqrt{\sum_{j=1}^{K}{\left(\mu_{j}c\right)}^{2}+\sigma_{j}^{2}\phi_{j}}}{\sum_{j=1}^{K}\phi_{j}},$$

along each cue dimension. Equations (7) and (8) calculate the central tendency and variability of the *mixture*, which is not distributed as a Gaussian. Thus, the mean and standard deviation may not accurately capture its properties. These are the same equations used by Toscano and McMurray [59] (though they were not specified explicitly in that paper). The values of *c* and *v* are then used to convert each cue input to a z-score-like value. For dimensions where the relative ordering of categories is reversed (e.g., /d/ has lower cue-values than /b/), category ordering is standardized by changing the sign of the normalized values when they are scaled. Thus, in these simulations, all normalized cue inputs are organized such that lower values are representative of /b/ and higher values are representative of /d/, providing a common reference frame for training the integrated representation.

To account for extreme cue-values (i.e., those that fall well outside the typical range along a given cue dimension), the normalized values are also scaled so that these values do not have undue influence on the input to the combined representation,

(9) $$x_{norm}=\frac{{\left(\frac{x-c}{v}\right)}^{t}}{s},$$

where *t* and *s* are scaling parameters. These values were arbitrarily set to 0.9 and 2.5, respectively, for all simulations presented here. Other methods (such as excluding extreme values) could have been used to deal this issue, but because we are generating the training values from actual distributions (and an extreme value can randomly occur occasionally), we did not want to eliminate such trials.

Normalized cue-values are then weighted based on the reliability of each cue, using the cue-weighting metric proposed by [59],

(10) $$w=\left(\sum_{n=1}^{K}\sum_{m=1}^{K}\frac{\phi_{m}\phi_{n}{\left(\mu_{m}-\mu_{n}\right)}^{2}}{\sigma_{m}\sigma_{n}}\right)÷2$$

This is the same basic measure as Equation (2), but generalized to allow for any number of components in the mixture by performing pairwise comparisons. As discussed in the Introduction, we cannot use the $1/\sigma^{2}$ metric described in cue-weighting models in vision, where the cue dimension is described by a unimodal distribution, because this would give incorrect estimates of cue reliability for phonetic cues. Instead, the category structure must be taken into account, as it is in this metric. (See [59,67] for further discussion of this issue.) Raw cue weights are normalized so they sum to 1, and the sum of the weighted cue-values is used as the input to train the integrated audiovisual mixture.

### 2.3. Learning Algorithms

Model parameters are updated via maximum likelihood estimation by gradient descent, which is an unsupervised statistical learning procedure. To derive the learning rules, we first take the log-likelihood of each mixture (Equation (6)) and compute the partial derivative of each Gaussian with respect to each parameter (*ϕ*, *μ*, *σ*),

(11) $$\Delta \phi_{j}=\eta_{\phi }\left(\frac{G_{j}\left(x\right)}{M\left(x\right)}\right)\left(\frac{1}{\phi_{j}}\right)$$

(12) $$\Delta \mu_{j}=\eta_{\mu }\left(\frac{G_{j}\left(x\right)}{M\left(x\right)}\right)\left(\frac{x-\mu_{j}}{\sigma_{j}^{2}}\right)$$

(13) $$\Delta \sigma_{j}=\eta_{\sigma }\left(\frac{G_{j}\left(x\right)}{M\left(x\right)}\right)\left(\sigma_{j}^{-3}{\left(x-\mu_{j}\right)}^{2}-\sigma_{j}^{-1}\right)$$

This applies to both the cue-level and integrated percept mixtures. $\eta_{\phi }$, $\eta_{\mu }$, and $\eta_{\sigma }$ are learning rates for each parameter. Learning rates for each mixture are given in Table 1. Note that learning rates differ between mixtures partly because the units are different for the auditory and visual cues; the learning rates used here were chosen to yield stable representations by the end of the training phase.

After updated model parameters are computed, winner-take-all competition is used to ensure that only the component with the largest Δϕ value has its *ϕ* parameter updated; all other values for Δϕ are set to 0. Because *ϕ* values along each dimension must sum to 1, the result is that the likelihood of the winning Gaussian will increase, and the others will decrease slightly. This competition mechanism allows the model to successfully eliminate components in the mixture that are not needed to capture the category structure of the language (by reducing their *ϕ*-values to near zero; see Figure 2b).

### 2.4. Training Data and Procedure

**Figure 3:**
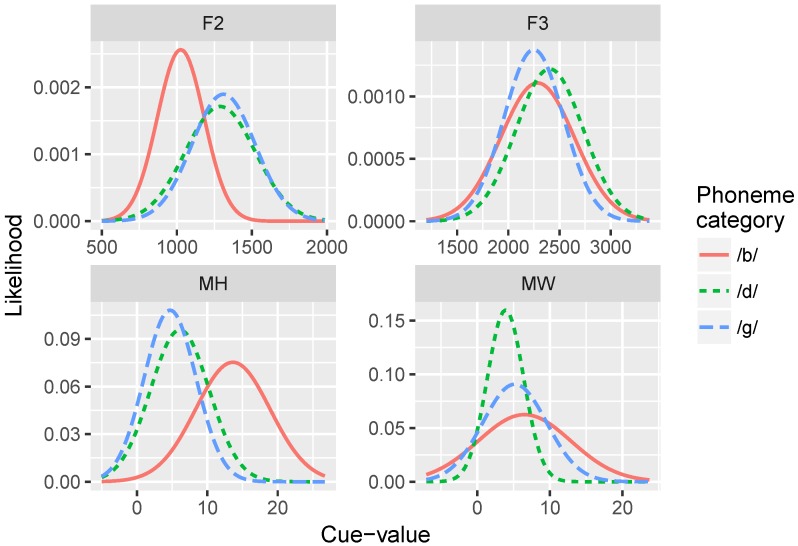
Statistical distributions for the four individual cues for the /b/, /d/, and /ɡ/ categories. Cue-values for F2 and F3 onset are in Hertz (Hz); cue-values for mouth height (MH) and mouth width (MW) are in millimeters (mm).

Five hundred repetitions of GMMs with four cue inputs were trained using data sampled from the cue distributions for the /b/-/d/ distinction and for the /b/-/ɡ/ distinction (see Table 2 and Figure 3). For each repetition, first an array of K=50 Gaussians was randomly generated to serve as the initial state. Initial *μ* values were randomly chosen from a normal distribution with a mean of the means of the two prototypes for that cue, and standard deviation of 3 for the visual dimensions, a standard deviation of 250 for F2, and a standard deviation of 350 for F3. Initial *σ* values were set to the mean of the standard deviations of the two prototypes for that cue. Initial *ϕ* values were all set to 1/K. After initializing the model, it was exposed to a set of inputs sampled from the cue distributions. On each trial, a set of visual and auditory values were selected, and the parameters of the Gaussians (*μ*, *σ*, and *ϕ*) were updated via the cue weighting and learning algorithms discussed above. This process was repeated for 100; 1000; 10,000; 100,000; and 150,000 trials.

### 2.5. Testing procedure

After training, the model was presented with values for each cue (MH, MW, F2, F3). Eighty-one test stimuli were derived from the cue distributions (see Table 2) including 9 auditory steps and 9 visual steps. For each cue, step 3 corresponds to the mean /b/ prototype value and step 7 corresponds to the mean /d/ (or /ɡ/) prototype value. Thus, steps where both auditory and visual cues are low are congruent /b/ values and steps where both auditory cues are high are congruent /d/ (or /ɡ/) values. For the congruency simulations, we used auditory step 1 paired with visual step 1 and auditory step 9 paired with visual step 9 as “congruent” stimuli and auditory step 1 paired with visual step 9 and auditory step 9 paired with visual step 1 as “incongruent” stimuli.

To convert model output into identification responses that correspond to data from human subjects, the posterior probability for each category was used to obtain of the proportion of /d/ (Simulation 1) or /ɡ/ (Simulation 2) responses using a Luce choice linking hypothesis [74],

(14) $$P\left(/d/|x\right)=\frac{G_{d}\left(x\right)}{G_{b}\left(x\right)+G_{d}\left(x\right)}$$

where *x* is the test stimulus, $G_{b}\left(x\right)$ is the component in the combined mixture with the highest posterior for the /b/ prototype, and $G_{d}\left(x\right)$ is the component with the highest posterior for the /d/ prototype. For Simulation 2, the posterior for the /ɡ/ prototype was used instead of /d/. This approach is similar to other models, such as FLMP.

## 3. Results

### 3.1. Simulation 1: */b/*-*/d/* Distinction

We first simulated the model’s ability to learn a /b/ vs. /d/ distinction based on the two visual cues (MH and MW) and two acoustic cues (F2 and F3). This allows us to evaluate whether statistical learning is sufficient for acquiring unified audiovisual perceptual representations, and whether the model shows the same developmental trajectory as human listeners (i.e., increased reliance of visual cues over the course of development, even in cases with incongruent audiovisual inputs).

#### 3.1.1. Training

First, we evaluated whether or not the training procedure was successful by assessing whether the model had at least a two-category representation in the combined (i.e., audiovisual) mixture at each of the four test times during training. Because the model learns by eliminating categories that are not needed to capture the data over the course of development, under-generalization is qualitatively different from over-generalization (which is a state the model typically cannot recover from given normal language inputs). Thus, we include in our analysis all model runs with representations for the integrated mixture that map cue prototypes onto two distinct categories for the relevant phonemes, even if there are additional above-threshold categories (i.e., categories with *ϕ*-values >1/K, the initial ϕ-value) at that point in development. The two categories selected that correspond to /b/ and /d/ are those that have the highest posterior for the category prototypes.

After 100; 1000; 10,000; 100,000 and 150,000 training trials, 99.8%, 100%, 99.6%, 99.0%, and 97.8% of the simulation runs converged on a solution with at least two categories. We can thus conclude that the model learned the /b/-/d/ phonemic distinction remarkably well, and that these basic statistical learning mechanisms allow the model to form unified perceptual representations that combine acoustic and visual cues. Note that the model does overgeneralize occasionally (2.8% of the simulations at the end of training), which is not indicative of typical development. However, this overgeneralization may simply be due to the fact that the learning rates and starting parameters for the model are not at the most stable point in parameter space (recall that we are not fitting model parameters to specific subjects’ data in these simulations).

**Figure 4:**
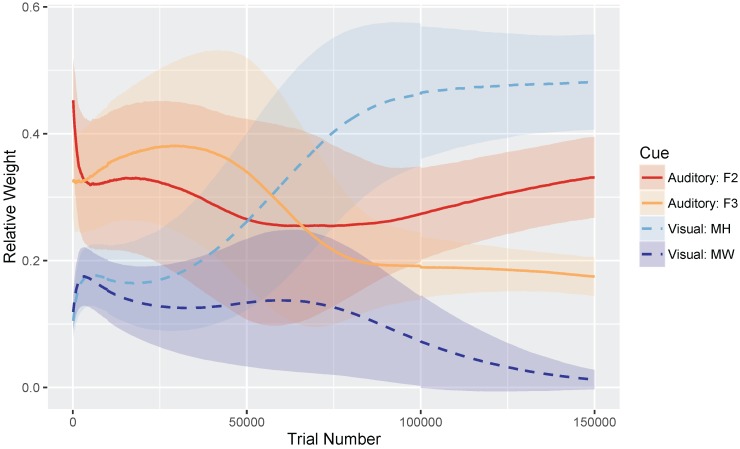
Simulation 1 (/b/-/d/ distinction): Normalized cue weights (mouth height, mouth width, F2 onset, F3 onset) over time with standard deviation error bars to show the spread across the 500 repetitions. By the end of training (i.e., trial 150,000), the cue weights are stable. The mouth width (MW) cue often overgeneralizes, resulting in a low relative weight.

Figure 4 shows the relative weights of the four cues (i.e., the sum of the four cues is equal to 1) as a function of trial number. Very early in development, after only 100 training trials, the model weights the auditory cues more heavily than the visual cues: mean weight of F2 =0.453 and F3 =0.323, whereas MW =0.119 and MH =0.105. At the end of training, corresponding to the point when the model has achieved a stable (i.e., adult-like) audiovisual representation, the model weights the cues by their reliability in distinguishing /b/ and /d/ (based on the overlap in the means and SDs for the category prototypes), assigning the greatest weight to the mouth height cue: MH =0.482, F2 =0.331, F3 =0.175, and MW =0.012.

We found that the MW cue sometimes overgeneralized to a one-category solution, which accounts for its low average relative weight at the end of training. This is a behavior consistent with the distributional statistics of that cue: MW provides very little information for distinguishing /b/ and /d/. As the cue distributions in Figure 3 show, the /b/ category is highly variable along this dimension, whereas /d/ is not. However, the means are very similar for the two phonemes. This is a particularly challenging representation for the model to stabilize on. This suggests that, in turn, human listeners would not rely on MW as a cue to this phoneme distinction—a hypothesis that would need to be tested.

#### 3.1.2. Identification Functions

**Figure 5:**
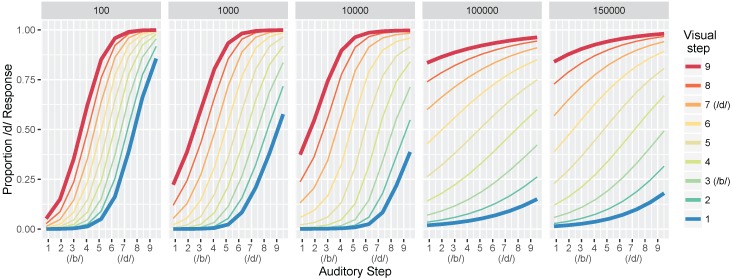
Simulation 1: model identification responses as a function of the nine auditory and nine visual steps and the total training trials (panels). Data are the mean proportion of /d/ responses averaged across 500 simulation runs of the model. The bolded red and blue lines are highlighted to correspond to the schematic predictions in Figure 1a; the 1000 panel is most closely matched to the “Children” panel and the 100,000 and 150,000 panels match the “Adult” panel.

Next, we computed identification functions from the model at each of our five test times. Figure 5 shows the mean proportion of /d/ responses at the five different levels of training for all 81 test stimuli (9 steps along the auditory continuum fully crossed with 9 steps along the visual continuum). Step 3 along both continua corresponds to the /b/ category prototype of our dataset (i.e., mean /b/ value), and step 7 is the /d/ prototype. We choose to highlight steps 1 and 9 because they more closely match the behavioral output reported by Massaro et al. [32].

Looking at the first panel (after 100 training trials), there is a robust effect of auditory cue-values on the model’s response: test values close to the *auditory* /b/ prototype produce mostly /b/ responses whereas test values close to the *auditory* /d/ prototype produce mostly /d/ responses. The same pattern is seen regardless of the *visual* value, as shown by the closeness of the colored lines. Thus, differences along the auditory dimensions produce large changes in the model’s identification of the phoneme category, while changes along the visual dimensions affect the model’s category judgments to a substantially smaller degree. With more training, the effect of visual cues on the model’s response becomes more pronounced, as shown by the increasing distance between the colored lines in the identification functions for 1000 and 10,000 trials. By 100,000 trials, test values close to the *visual* /b/ prototype (i.e., the blue line) produce mostly /b/ responses whereas test values close to the *visual* /d/ prototype (i.e., the red line) produce mostly /d/ responses, regardless of the *auditory* value.

Overall, these results are consistent with observations from human listeners, who show increased effects of visual cues in trading relations paradigms (like the one used here to test the model), as a function of age (as in Figure 1a). Indeed, despite not fitting the model to perceptual data, the range of responses produced at different points in training mirror responses observed with children and adults in Massaro et al. [32].

#### 3.1.3. Congruency Effects

**Figure 6:**
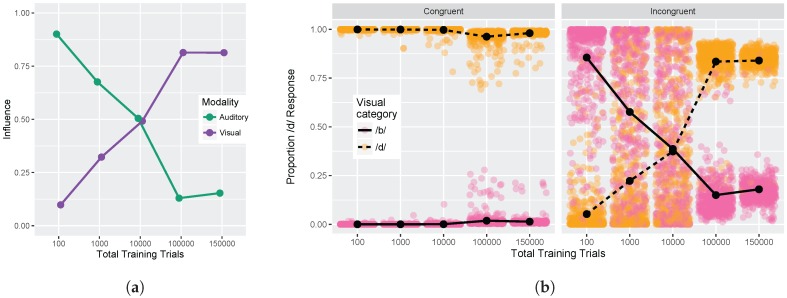
Simulation 1: Congruency effect. (**a**) Change in auditory and visual influence over development: visual influence increases whereas auditory influence decreases (as in the schematic Figure 1b); (**b**) Left panel displays model responses for congruent stimuli, and right panel displays responses for incongruent stimuli. Individual data points reflect model responses on each of the 500 runs of the stimulation (position jittered to avoid overplotting). Lines represent mean response across the 500 simulation runs. Data are plotted as the proportion of /d/ responses as a function of the *visual* stimulus.

Finally, we examined model responses as a function of whether visual and auditory stimuli were congruent or incongruent. We used auditory and visual step 1 and step 9 from Figure 5 (i.e., the endpoints of the red and blue lines) for this analysis. These values were used to compute the auditory and visual influence at each training time (see Equations (3) and (4)). Figure 6a shows the change in auditory and visual influence over development: visual influence increases, whereas auditory influence decreases, consistent with the behavioral data (see Figure 1b and [32,33,34,35,36,37]).

**Figure 7:**
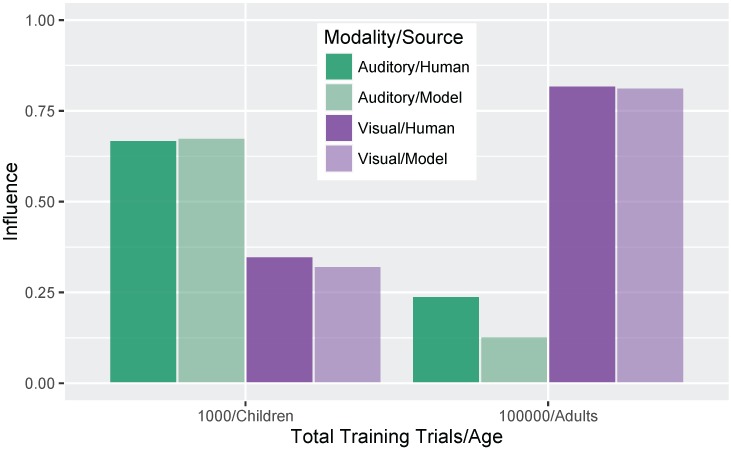
Simulation 1: Auditory and visual influence comparison between the model results (at 1000 and 100,000 trials) and behavioral results from Massaro et al. [32].

Figure 7 shows a more direct comparison to the behavioral results reported in Massaro et al. [32]. Although there is no specific mapping between the model’s training trials and human age, we were able to identify two points where the absolute size of the auditory and visual influence in the model was close to the child and adult data reported by Massaro and colleagues (1000 and 100,000 training trials, corresponding to children and adults, respectively). For children, Massaro et al. [32] reported an auditory influence of 0.67 and visual influence of 0.35. At the corresponding stage of development, the model shows a very close match to those values with an auditory influence of 0.68 and visual influence of 0.32. For adults, Massaro et al. [32] reported an auditory influence of 0.24 and visual influence of 0.82; similar values are found for the adult-like model, though the size of the auditory influence is somewhat smaller: auditory influence is 0.13 and visual influence is 0.81. Thus, the model was able both to capture the change over development and match the size of the auditory and visual influence for children and adults.

Figure 6b provides an alternative way of looking at the effects of congruency at each of our five test times. The individual points represent the results of the 500 simulation runs (which can be interpreted similarly to individual differences between subjects). The *Congruent* panel shows the proportion of /d/ responses when the auditory and visual inputs provide redundant information (i.e., pink points indicate that auditory and visual inputs both signal /b/ and orange points indicate that auditory and visual inputs both signal /d/). The model is almost perfect in its phoneme categorization for congruent stimuli; mean accuracy at each test time was 99.9%, 99.9%, 99.8%, 97.2%, and 98.3%.

The *Incongruent* panel shows the proportion of /d/ responses when the auditory and visual inputs provide mismatching information (i.e., pink points indicate that visual cue-values signal /b/ while auditory cue-values signal /d/, and vice versa for orange points). Early in development (after 100 training trials), the model largely ignores the conflicting visual input in favor of the auditory cues. After 1000 training trials, the model responses are already less biased towards auditory inputs, and after 10,000 training trials, model responses are evenly split between auditory-based and visual-based responses. By 100,000 and 150,000 training trials, the model is heavily biased to produce a response consistent with the visual input, largely ignoring the conflicting auditory cues. At each time point, there are some pink/orang points that cross the midpoint, which is representative of individual trials (or listeners) who do not show the dominant pattern.

### 3.2. Simulation 2: */b/*-*/ɡ/* Distinction

To determine whether the effect holds for /b/-/ɡ/ distinctions, we ran a second set of simulations with cue distributions corresponding to those phonemes. All other simulation parameters were the same as above.

#### 3.2.1. Training

First, as above, we evaluated whether the model was successful in learning the /b/-/ɡ/ contrast (i.e., whether it maintained at least a two-category representation in the audiovisual mixture) at each of the five test times. After 100; 1000; 10,000; 100,000 and 150,000 training trials, 100%, 99.8%, 100%, 98.4%, and 98.8% of the simulation runs converged on a solution with at least two categories. The success rate here was comparable to Simulation 1; thus we can conclude that the model learned the /b/-/g/ phonemic distinction well.

**Figure 8:**
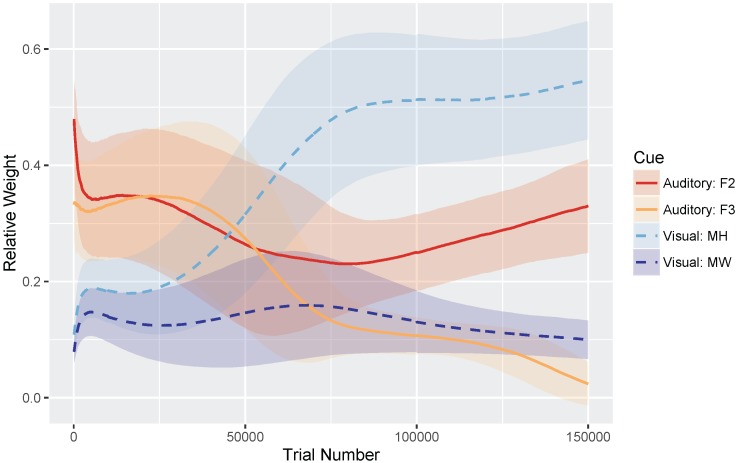
Simulation 2 (/b/-/ɡ/ distinction): Normalized cue weights (mouth height, mouth width, F2 onset, F3 onset) over time with standard deviation error bars to show the spread across the 500 repetitions. The mouth width (MW) and F3 cues often overgeneralized by the end of training (i.e., trial 150,000), resulting in a low relative weight.

Figure 8 shows the relative weights of the four cues. As in Simulation 1, after only 100 training trials, the model weights the auditory cues more heavily than the visual cues: mean weight of F2 = =0.480 and F3 =0.333, whereas MW =0.079 and MH =0.108. At the end of training, model weights more closely correspond to the relative reliabilities of the cues for the /b/-/ɡ/ distinction: MH =0.556, F2 =0.330, MW =0.100, and F3 =0.024. Similar to Simulation 1, MW often overgeneralized here; additionally F3 also tended to overgeneralize by the end of training. This pattern is consistent with the cue distributions (Figure 3) for the /b/-/ɡ/ distinction.

#### 3.2.2. Identification Functions

**Figure 9:**
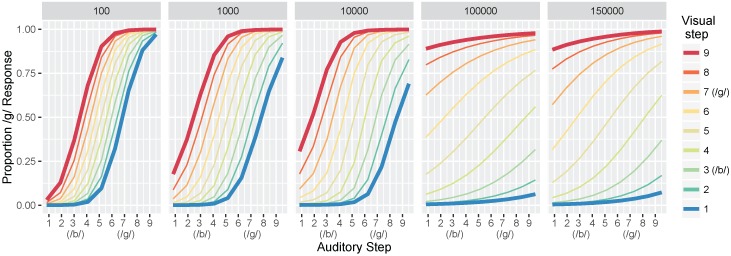
Simulation 2: model identification responses as a function of step along the auditory continuum and step along the visual continuum. The results are similar to Simulation 1 (Figure 5).

Next, we computed identification functions at the five test times, as in Simulation 1. Figure 9 shows the mean proportion of /ɡ/ responses as a function of differences along the auditory and visual continua. After 100 trials, we see a similar pattern to that of the previous simulation, with responses primarily determined by the acoustic cues. The effect of the visual cues appears a bit slower to develop, but is even more robust at the end of training (150,000 trials) than in the /b/-/d/ simulation, suggesting a potentially larger role for visual information for the /b/-/ɡ/ contrast. These results are again consistent with observations from human listeners.

#### 3.2.3. Congruency Effects

**Figure 10:**
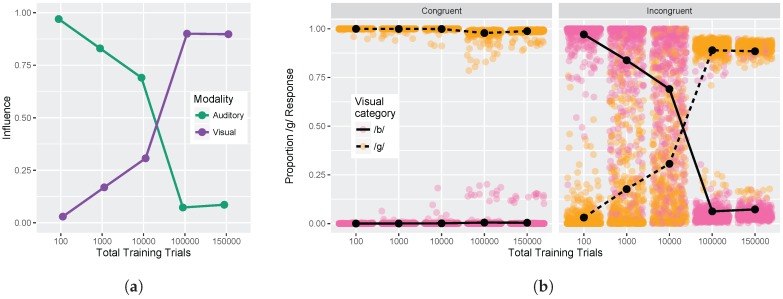
Simulation 2: Congruency effect. (**a**) Change in auditory and visual influence over development: visual influence increases whereas auditory influence decreases (as in the schematic Figure 1b and Simulation 1 Figure 6a); (**b**) Left panel displays model responses for congruent stimuli, and right panel displays responses for incongruent stimuli. Lines represent mean response across the 500 simulation runs. Data are plotted as the proportion of /d/ responses as a function of the *visual* stimulus (as in Figure 6b).

Finally, we examined the congruency effect for the /b/-/ɡ/ contrast. As shown in Figure 10a, the model shows a change from more auditory influence after 100 training trials to more visual influence after 100,000 and 150,000 training trials. As in Simulation 1, the effect emerges over the course of development in a manner consistent with changes observed between infants, children, and adults.

Figure 10b shows model responses to congruent and incongruent stimuli at each of our five test times. The model showed almost perfect categorization in the congruent condition; mean accuracy at each test time was 99.9%, 99.9%, 99.9%, 98.6%, and 99.2%. In the incongruent condition, the model largely relies on auditory cues at the earliest point in development, just as it did for the /b/-/d/ contrast. Model responses are not evenly split between auditory-based and visual-based responses by 10,000 trials as in Simulation 1, but rather remain slightly biased towards auditory responses overall. However, by 100,000 and 150,000 trials, the model produces responses consistent with the visual cues a majority of the time.

## 4. Discussion

We sought to investigate whether unsupervised, competitive, statistical learning mechanisms are sufficient for acquiring unified audiovisual perceptual representations. We also asked whether the model would show the same developmental trajectory as human listeners in terms of changes in its use of visual cue information (i.e., increased reliance of visual cues over the course of development), including changes in model responses to congruent and incongruent audiovisual stimuli.

These hypotheses were supported by the model simulations. The simulations demonstrate that weighting-by-reliability can be used to successfully describe the developmental trajectory of audiovisual speech learning. Early in training, the model weights the two auditory cues higher than the two visual cues. Later in development, when the model has achieved a stable (i.e., adult-like) representation, it successfully weights the cues by their reliability in distinguishing the trained phonemes. When testing the model at various stages of development, we found that it showed increased effects of visual cues as a function of age, which is consistent with observations from human listeners: children show a much smaller effect of visual speech cues on categorization than adults do [12,32]. Further, we found that audiovisual congruency effects emerged over the course of learning, such that the model initially makes responses consistent with the auditory stimulus and then switches to visual-based responses with additional training. This is again consistent with what we observe with human infants and children [33,34,35,36,37], with the size of the auditory and visual influence in the model closely matching the effect sizes seen in data from children and adults [32].

Thus, we provide an existence proof that statistical learning, as implemented in the GMM [52,59], can be used to acquire unified audiovisual representations that follow the same developmental trajectory as human learners and produce categorization functions that mirror those obtained in behavioral experiments. This is significant because it provides a learning mechanism that makes few assumptions about the information available to infants and children. The learning algorithm and cue-weighting metric used in the model make no *a priori* assumptions about the number of phonological categories, their distributional statistics, or the reliability of the cues. As the model learns, it weights and integrates inputs from each cue according to their reliability in order to learn the integrated audiovisual distinction between stop consonants. This learning process takes place iteratively, and at no point during training do we need to present labeled inputs to the model about which category is “correct”—these distinctions can all be learned using the same types of statistical learning mechanisms available to human infants.

This is also the first time GMM models have been used for visual inputs in addition to acoustic cues. The results suggest that the emergence of unified perceptual representations and phenomena like the McGurk effect do not require specialized learning mechanisms, though we cannot rule out the possibility that infants use additional mechanisms as well (the current results simply suggest that additional mechanisms are not necessary). Both auditory and visual speech cues can be represented as statistical distributions, putting them into a common representational frame that can then be used to acquire audiovisual speech categories.

It is worth noting that our model shows similar results regardless of audiovisual pairing (i.e., auditory /ɡ/ + visual /b/ is similar to auditory /b/ + visual /ɡ/), whereas studies of the original McGurk effect found an asymmetry depending on the pairing (i.e., auditory /ɡ/ + visual /b/ shows more auditory-bias than auditory /b/ + visual /ɡ/, which results in the fusion response /d/). However, the original effect [10,11] was generated by asking participants to repeat what they heard in a task with an open-ended response. Therefore, our model is more similar to the two-alternative forced-choice task used by Massaro and colleagues [32,35,62], and our results match nicely with the behavioral data they present.

Several of our model implementation choices deserve further discussion. Most importantly, we did not run a simulation including a three-way distinction between /b/, /d/, and /ɡ/. Although this would allow a closer comparison to the original McGurk effect [10], it was unrealistic given the dataset we had available in these simulations [72]. Consider again the cue distributions shown in Figure 3: notice that for the majority of the cues we chose, a three-way distinction is not obvious from the distributions. Even for the more reliable cues (F2 and MH), there seems to be a /b/ vs. non-/b/ category distinction, but not a /d/ vs. /ɡ/ distinction. However, the model could, in principle, be trained on a three-way distinction if a set of non-overlapping cue distributions existed with the same relative category order for each cue. The cue weighting metric is also generalizable to three-way distinctions. Thus, this is not a limitation of the model, *per se*, but rather a limitation of the input data that necessitates our separate simulations for the /b-d/ and /b-g/ distinctions. In addition, several of the comparisons we make with data from human subjects rely on two-way distinctions, rather than a three-way /b/-/d/-/g/ contrast.

Second, previous simulations using the GMM [52,59] did not include the scaling parameters for the normalized inputs (Equation (9)) to account for extreme training values along the cue dimensions. We ran a set of 100 simulation runs without the parameters (i.e., with *t* =1 and *s* =1) for the /b/-/d/ distinction. The combined audiovisual mixture was not more likely to overgeneralize without these parameters (99% arrived at at least a two-category solution); however the inclusion of these parameters has merit developmentally. A human listener who hears or sees extreme input values (e.g., over-exaggerated mouth movements) would either ignore that instance or scale it into a more plausible value given the rest of its input (i.e., assign it to the closest matching phoneme category).

Third, we set the *ϕ* learning rates ($\eta_{\phi }$) for the visual cues to be slower in comparison to the learning rates for the auditory cues (Table 1). To confirm that this choice alone did not drive the pattern of results observed, we ran a set of 100 simulation runs with the auditory learning rates slowed down to be equal to the visual learning rates ($\eta_{\phi }=0.00005$ for all four cues) for the /b/-/d/ distinction. The combined audiovisual mixture overgeneralized slightly more often by 150,000 trials (93% converged on at least a two-category solution). This led to a more equal proportion of /b/ and /d/ responses for incongruent test stimuli than the simulations with faster auditory learning rates. This difference suggests that it is not the absolute learning rates impacting the model’s performance, but the *relative* rate difference; the combined mixture overgeneralizes if all four cue rates are the same, but largely achieves a two-category solution if the visual cue learning rates are slower than the auditory rates. Indeed, it makes sense developmentally that visual cues would take longer to acquire than auditory cues, as infants are likely to have more learning opportunities on the basis of auditory input alone (i.e., they only get visual cues if they are looking at the talker).

Finally, although the initial cue weights (before training) were approximately equal across the four cues, as determined by the spread of the Gaussians in each mixture at the start of the simulation, there was a bias towards higher average weights for the auditory cues than the visual cues for the starting parameters we used. Although the selection of starting values is somewhat arbitrary, it was important to verify that our simulation outcome was not just a product of poor initial values. To test this, we updated the spread of the initial *μ* values for each cue to ensure that the initial weights were exactly the same (i.e., within 0.9% of each other), and ran a set of 100 simulation runs on the /b/-/d/ distinction. Despite closer relative cue weights between the four cues at trial 100, the testing results still showed an increase in the use of visual cues over the course of development, though the model was more likely to overgeneralize (89% converged on at least a two-category solution). This suggests that an early auditory bias is not needed to to show these effects, removing one additional assumption about what is needed for the effects to emerge.

## 5. Conclusions

We presented a model of phonetic category acquisition trained on data derived from measurements of visual and auditory speech cues for stop consonants. The model uses the same statistical learning processes available to human infants and shows the same developmental trajectory in its use of auditory and visual cues for speech categorization. We conclude that such processes provide a viable mechanism for explaining the acquisition of integrated audiovisual percepts.

## Figures and Tables

**Table 1:** Learning rates ($\eta_{\mu }$, $\eta_{\sigma }$, and $\eta_{\phi }$) for mouth height, mouth width, F2, F3, and the combined mixture.

Cue	$\eta_{\mu }$	$\eta_{\sigma }$	$\eta_{\phi }$
Mouth width	0.009	0.008	0.00005
Mouth height	0.009	0.008	0.00005
F2 onset	40	20	0.0005
F3 onset	80	40	0.0005
Integrated	0.00001	0.00001	0.0005

**Table 2:** Means (standard deviations) for each cue (mouth height, mouth width, F2, F3) for the tested phonological categories (/b/, /d/, /ɡ/) measured from the Audio-Video Australian English Speech (AVOZES) corpus Goecke [72].

Cue	/b/	/d/	/ɡ/
Mouth width (range, mm)	6.51 (6.39)	3.89 (2.50)	5.16 (4.40)
Mouth height (range, mm)	13.63 (5.30)	6.09 (4.17)	4.70 (3.69)
F2 onset (minimum, Hz)	1026 (155.70)	1289 (232.80)	1311 (210.30)
F3 onset (minimum, Hz)	2286 (360.16)	2404 (326.98)	2249 (290.26)
